# The relationship between urinary iodine concentration and papillary thyroid cancer: A systematic review and meta-analysis

**DOI:** 10.3389/fendo.2022.1049423

**Published:** 2022-10-31

**Authors:** Xueqi Zhang, Fan Zhang, Qiuxian Li, Renaguli Aihaiti, Chuyao Feng, Deshi Chen, Xu Zhao, Weiping Teng

**Affiliations:** Department of Endocrinology and Metabolism, Institute of Endocrinology, National Health Commission Key Laboratory of Diagnosis and Treatment of Thyroid Diseases, The First Hospital of China Medical University, Shenyang, Liaoning, China

**Keywords:** urinary iodine concentration, iodine, iodine nutrition, papillary thyroid cancer (PTC), meta-analysis

## Abstract

**Background:**

The effect of iodine on papillary thyroid cancer (PTC) has been controversial for many years. Since urinary iodine is an effective indicator of iodine intake, some recent epidemiological studies have described the relationship between urinary iodine concentration (UIC) and PTC.

**Methods:**

We searched PubMed, Embase, Cochrane Library, and Web of Science for case-control studies about UIC and PTC published before September 2022. Results are presented as the overall odds ratio (OR) and 95% confidence intervals (CI).

**Results:**

According to the analysis of the included studies, excessive iodine intake (UIC≥300ug/L) was positively associated with the occurrence of PTC patients compared with healthy controls (OR4.05, 95%CI 1.64-10.02, P=0.002). Meanwhile, adequate iodine exposure (100≤UIC<200ug/L) may play a protective role in the occurrence of PTC compared with healthy individuals (OR 0.36, 95%CI 0.14-0.91, P=0.03) while the difference in the prevalence of insufficient iodine intake (UIC<100ug/L) and iodine above requirements (200≤UIC<300ug/L) among the two groups were not significant (deficiency: OR 0.38, 95%CI 0.13-1.16, P=0.09; above requirements: OR 0.92, 95%CI 0.40-2.10, P=0.84). After comparing the UIC levels of PTC patients with those of other thyroid diseases, we found that there was also no significant difference in the incidence of different levels of UIC in the two groups (excessive: OR 1.25, 95%CI 0.87-1.80, P=0.22; above requirements: OR 0.93, 95%CI 0.77-1.14, P=0.49; adequate: OR 0.96, 95%CI 0.78-1.17, P=0.67; deficiency: OR 1.02, 95%CI 0.86-1.22, P=0.80). The result of this meta-analysis also did not support the relationship between UIC and the BRAF mutation and lymph node metastasis (LNM) of PTC patients. Besides, we also found that studies on the relationship between urinary iodine and PTC may be influenced by the way UIC was measured.

**Conclusion:**

The 10 case-control included studies involved a total of 6,544 participants. The results of this meta-analysis showed excessive iodine intake, that is, UIC≥300ug/L was associated with the occurrence of PTC but not with BRAF mutation and LNM while adequate iodine intake (100≤UIC<200ug/L) may be one of the protective factors for PTC.

## Introduction

Since the 1990s, the incidence of thyroid cancer (TC) especially papillary thyroid carcinoma (PTC) has continued to increase worldwide (1, 2). Therefore, numerous experts around the world have explored the causes of this phenomenon. Risk factors including radiation exposure, dietary nutrition, environmental pollutants, family history, and overdiagnosis have been reported by many previous studies (3, 4). Among these factors, iodine is an important trace element that is closely related to thyroid function, it has also become a focus and has been debated for many years because of numerous inconsistent epidemiological findings. As a crucial micronutrient and a vital composition for the biosynthesis of thyroid hormone, iodine plays an important part in biochemical and metabolic pathways throughout the human body (5). At the same time, iodine status is a primary determinant of thyroid disorders (6), a U curve has been verified by many studies, which means that iodine deficiency or excess can lead to thyroid dysfunction (7, 8).

Through extensive literature review, we found that many epidemiological studies assessed iodine intake by regional water iodine (9), consumption of iodized salt (10), vegetable intake (11), and seafood intake of residents (12–14) and verified that iodine nutrition may be an important factor for the development of PTC. Due to the different methods of iodine nutrition detection used in these above studies on the association between iodine nutrition and PTC, the conclusions they obtained have great inconsistency and are difficult to interpret. During these years, many epidemiological studies have assessed the relationship between PTC and iodine nutrition based on the measurement of urinary iodine concentration (UIC) for the reason that urine is the main excretion route of ingested iodine (15, 16). The median UIC has also been considered an excellent biomarker of recent exposure to iodine in populations (17). According to WHO iodine recommendations, the status of iodine nutrition can be divided into four stages: UIC<100µg/L(insufficient), 100–199µg/L(adequate),200–299µg/L (above requirements), and ≥300 µg/L (excessive) (17, 18). Though many studies were affected by many factors such as ethnic differences, and diet customs (19), some researchers have found that different UIC levels may affect the occurrence of PTC. In addition, there have also been some epidemiological studies on the relationship between UIC and the invasiveness of PTC such as BRAF mutation (20) and lymph node metastases (LNM). However, the correlation between UIC and these alterations in PTC patients remains controversial (21).

Due to the limited conditions of most studies, the observed evidence is not sufficient, it is necessary to combine those similar studies, which may help to find the real answer to the controversial conclusions. Therefore, we collected case-control studies that investigated the relationship between UIC and PTC, which can find an answer about the correlation between iodine nutrition and PTC, and provide a reasonable, secure UIC range.

## Materials and methods

### Search strategy

**Figure 1:**
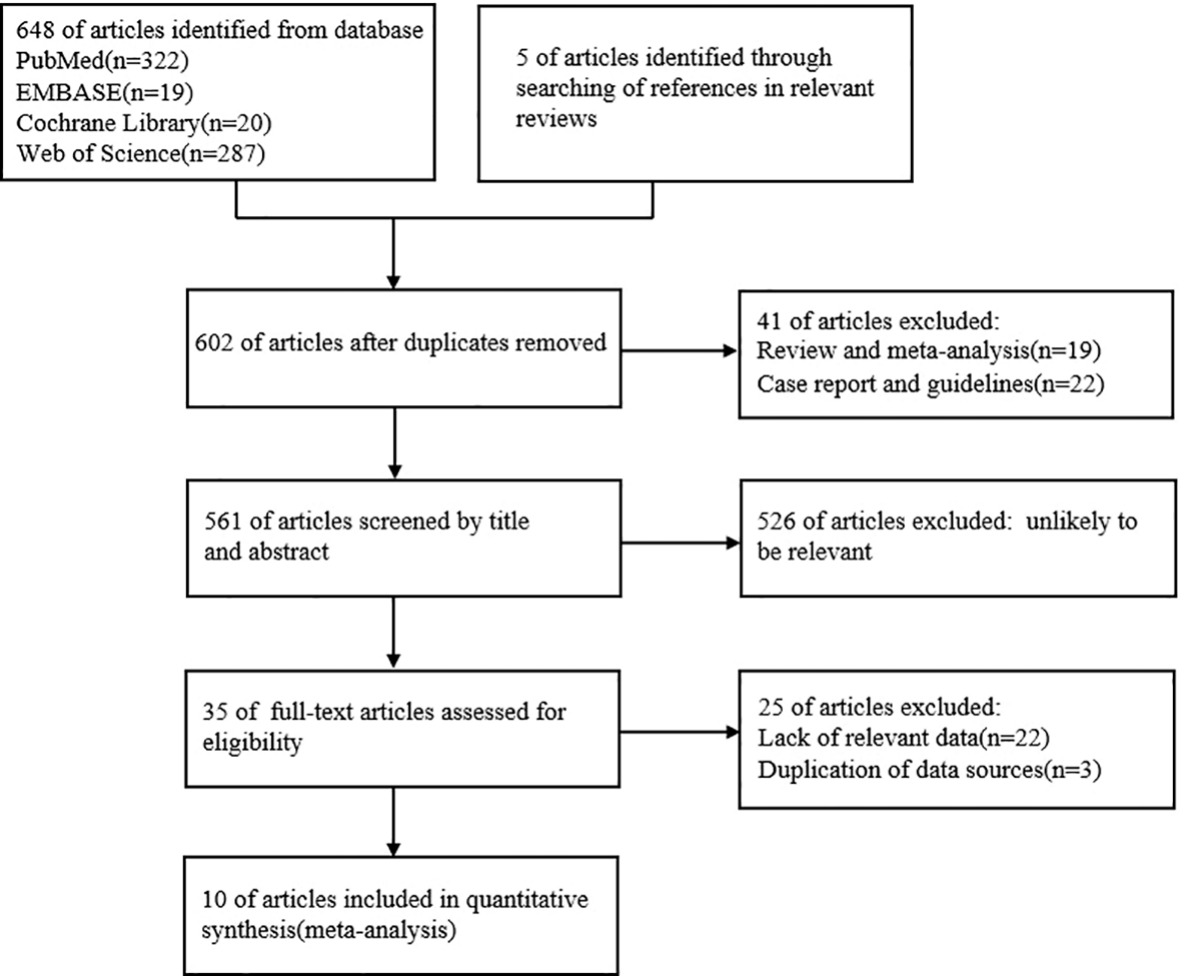
Flow chart of the inclusion or exclusion procedure.

We investigated the relationship between UIC and PTC by conducting a systematic literature search in PubMed, Embase, Cochrane Library, and Web of Science databases until September 2022. The search was conducted by using the search terms “thyroid cancer, papillary”; “Case-Control Studies” with the following keywords: iodine; iodine nutrition; iodine intake, and urinary iodine concentration. We selected relevant articles by classifying the title, abstract, and full text of all the studies and limiting the included studies to those published in English. Two researchers independently selected articles and reviewed the abstracts and full text of the articles. By searching PubMed (n=322), Embase (n=19), Cochrane Library (n=20), Web of Science(n=287), and references in relevant reviews(n=5), titles and abstracts of 561 articles were reviewed, 35 articles related to the topic were read in full. The process is described in a flow chart (**Figure 1**). After all these exclusion steps, 10 studies were included in this meta-analysis.

### Inclusion and exclusion criteria

Studies were considered eligible if they met the following criteria (1): urinary iodine levels were investigated and the patient was diagnosed with PTC (2). data on UIC were provided (3). participants were divided into PTC and non-PTC patients(other types of thyroid diseases including patients with benign thyroid nodules, nodular goiter, and thyroid adenomas), PTC patients with LNM or without lymph node metastases (NLNM), or PTC patients with or without BRAF mutation (4). written in English. In contrast, studies were excluded if they met one of the following criteria (1): the article was a case report, review, editorial, letter, or animal model subjects (2). missing relevant data or information (3). duplicate reports of the same original data. The selection process was completed by two authors independently, and differences were resolved through consultation or discussion with the third author.

### Data extraction

The following data were extracted (1): basic information (first authors, year of publication, study period, and location) (2); participant characteristics (sample size, number of cases, and controls) (3); the measured time and method of UIC. Two reviewers organized the extracted data, cross-checked the data, and assessed the quality of the included studies based on the Newcastle-Ottawa Quality Assessment Scale, which includes the selection of case and control groups, comparability, and exposure.

The total score ranged from 6 to 9, with high and low scores judging the quality of the research. All discrepancies or inconsistencies were resolved by consensus with the third author.

### Statistical analyses

Review-Manager 5 software (Copenhagen: The Nordic Cochrane Centre. The Cochrane Collaboration) was applied to all analysis processes. Odds ratios (ORs) and the corresponding 95% confidence intervals (95% CIs) were calculated to assess the strength of the association between UIC and PTC. The significance of the combined OR was determined by the Z test, and a P value < 0.05 was considered significant. We used the Cochran Q test (P < 0.05 indicated statistical significance) and the I^2^ statistic to assess heterogeneity. I^2^ values of 25%, 50%, and 75% were related to low, moderate, and high heterogeneity, respectively. When heterogeneity was high (I^2^>50%), a random-effects model was used to pool the results. In addition, if the heterogeneity was low, the fixed-effects model was used. To assess the stability of the results, we performed a sensitivity analysis by omitting one report at a time and recomputing the pooled estimates of the remaining studies. Funnel plots were constructed to assess publication bias.

## Results

### Characteristics of the included studies

We searched relevant literature through PubMed, Embase, Cochrane Library, Web of Science databases, and references in relevant reviews until September 2022, articles published in September 2022 are not included (manuscript submission: 20 September). A total of 653 records were identified from the initial search. A total of 602 articles remained after removing duplicate records, 35 articles were selected for further evaluation by screening titles and abstracts. A total of 10 original studies were finally included in the systematic review and meta-analysis after reading the full text and applying the inclusion and exclusion criteria (**Figure 1**).

The key characteristics of the studies involved in this meta-analysis are summarized in **Table 1**. Overall, the pooled data from 10 studies included 6,544 participants, distributed in China, Korea, and Turkey. The sample size of the included studies ranged from 132 to 2,041. 5 of these articles explored the level of UIC in PTC patients versus healthy controls (22, 26, 29–31), 7 of them examined UIC in PTC patients versus non-PTC patients(including patients with benign thyroid nodules, nodular goiter, and thyroid adenomas) (22, 24–27, 30, 31), 3 of them studied the relationship between UIC and BRAF mutation in PTC patients (23, 25, 29), and 3 cases examined the relationship between UIC and LNM in patients with PTC (23, 28, 29). We divided urinary iodine into four categories according to the data in these articles. Excessive iodine intake: UIC≥300ug/L, iodine above requirements: 200≤UIC<300ug/L, iodine in moderation:100≤UIC<200ug/L, and iodine deficiency: UIC ≤ 100ug/L. By extracting and analyzing the data of UIC among the participants, we hope to answer some controversial questions about iodine nutrition and PTC.

**Table 1:** Characteristics of the included studies in the meta-analysis.

No.	First author	Publication year	Study period(year/month)	Location	Sample size (n)	PTC patients	Non-PTC individuals	Urine samples	Measurement of UIC	Score
1	Wang (22)	2014	2010/06-2011/06	China	460	103 PTC patients	306 healthy individuals; 51 BTN patients	Morning urine	As-Ce catalytic spectrophotometry	8
2	Kim (23)	2017	2010/11-2015/03	Korea	215	215 PTC patients	–	Fasting, spot-urine	ICP-MS	8
3	Zhao (24)	2018	2013/11-2015/03	China	2041	1120 PTC patients	921 NG patients	Spot urine	As-Ce catalytic spectrophotometry	7
4	Celik (25)	2020	2015-2018	Turkey	132	88 PTC patients	44 BTN patients	Spot urine in the morning	As-Ce catalytic spectrophotometry	6
5	Hou (26)	2020	2017/01-2019/03	China	488	214 PTC patients	82 healthy individuals; 192 BTT patients	Random spot urine	As-Ce catalytic spectrophotometry	9
6	Huang (27)	2020	2019/07-2019/12	China	151	97 PTC patients	54 BTN patients	Fasting urine in the morning	As-Ce catalytic spectrophotometry	7
7	Fan (28)	2021	2015/03-2018/03	China	402	402 PTC patients	–	Urine after fasting and avoiding alcohol and medicine for 12 h	As-Ce catalytic spectrophotometry	7
8	Kim (29)	2021	2010/04-2014/12	Korea	946	446 PTC patients	500 healthy individuals;	Urine after an 8-hour fast	ICP-MS	9
9	Yu (30)	2021	2016/01-2020/12	China	1341	285 PTC	874 healthy individuals; 745 other thyroid diseases	Random spot urine	As-Ce catalytic spectrophotometry	8
10	Wang (31)	2022	2018/09-2019/09	China	368	112 PTC patients	184 healthy individuals; 72 BTN patients	Morning fasting middle-segment urine	ICP-MS	7

### UIC in PTC patients vs. healthy controls

**Figure 2:**
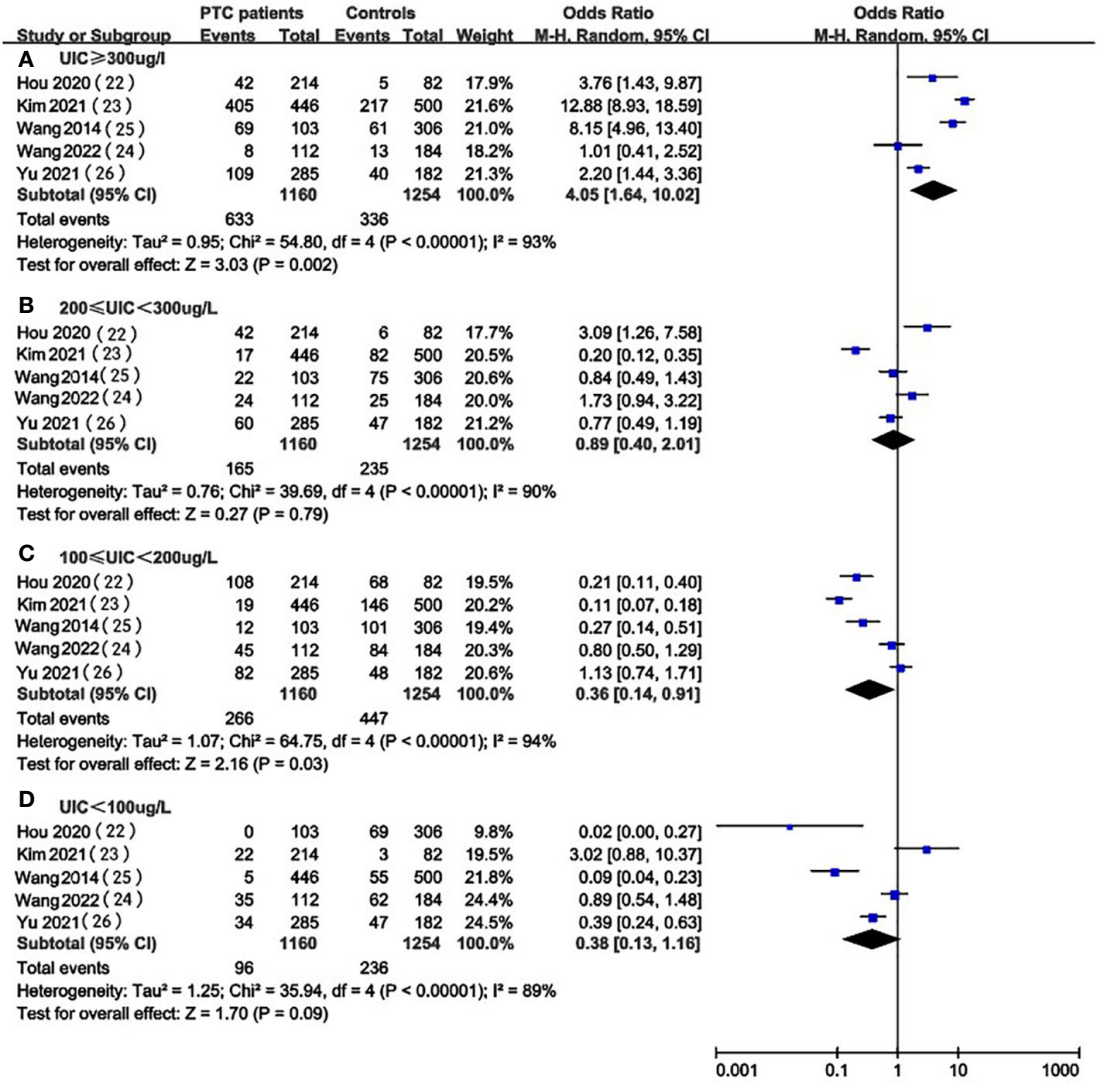
A between-group meta-analysis of UIC in PTC patients versus healthy individuals (controls).

In the pooled results of 5 studies (22, 26, 29–31), the prevalence of excessive iodine intake(UIC≥200ug/L) was statistically significant (OR 4.05, 95%CI 1.64-10.02 P=0.002, n = 2,414) (**Figure 2A**), which indicated that higher level of UIC may play a role in the prevalence of PTC. The results also indicated that adequate iodine intake(100≤UIC<200ug/L) was more common in healthy individuals than in patients with PTC (OR 0.36, 95%CI 0.14-0.91, P=0.03, n=2,414) (**Figure 2C**), which indicated that appropriate iodine nutrition may be one of the protective factors for PTC. However, the analysis result of iodine deficiency (UIC<100ug/L) (OR 0.38, 95%CI 0.13-1.16, P = 0.09, n = 2,414) (**Figure 2D**) and mild excess (200≤UIC<300ug/L) (OR 0.89, 95%CI 0.40-2.01, P = 0.89, n = 2,414) (**Figure 2B**) were not statistically significant so we cannot predict the exact effect of iodine deficiency and above requirements on PTC occurrence.

### UIC in PTC patients vs. non-PTC patients

**Figure 3:**
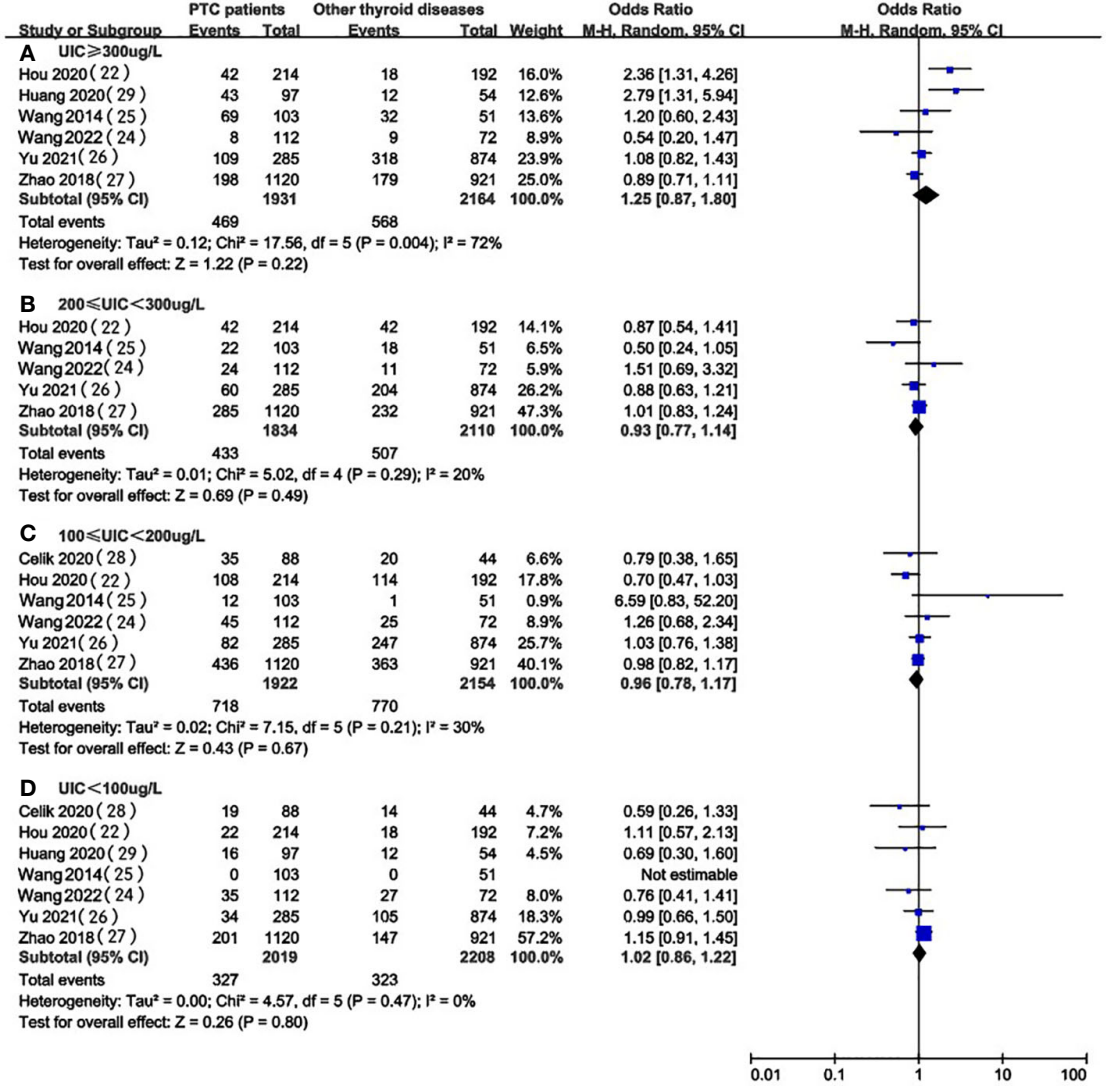
A between-group meta-analysis of UIC in PTC patients versus patients with non-PTC patients (controls).

**Figure 3** provides the forest plots of the relationship between UIC and the occurrence of PTC compared with non-PTC patients. In the pooled results of 7 studies (22, 24–27, 30, 31), there was no significant difference in UIC level between PTC patients and patients with other thyroid diseases (excessive: OR 1.25, 95%CI 0.87-1.80, P = 0.22, n=4,095; above requirements: OR 0.93, 95%CI 0.77-1.14, P=0.49, n=3,944; adequate: OR 0.96, 95%CI 0.78-1.17, P = 0.67, n = 4,076; insufficient: OR 1.02, 95%CI 0.86-1.22, P=0.80, n = 4,227) (**Figure 3**). Therefore, we could not indicate an association between UIC and the prevalence of PTC when compared with non-PTC patients.

### UIC in BRAF mutation vs. non-BRAF mutation in PTC patients

**Figure 4:**
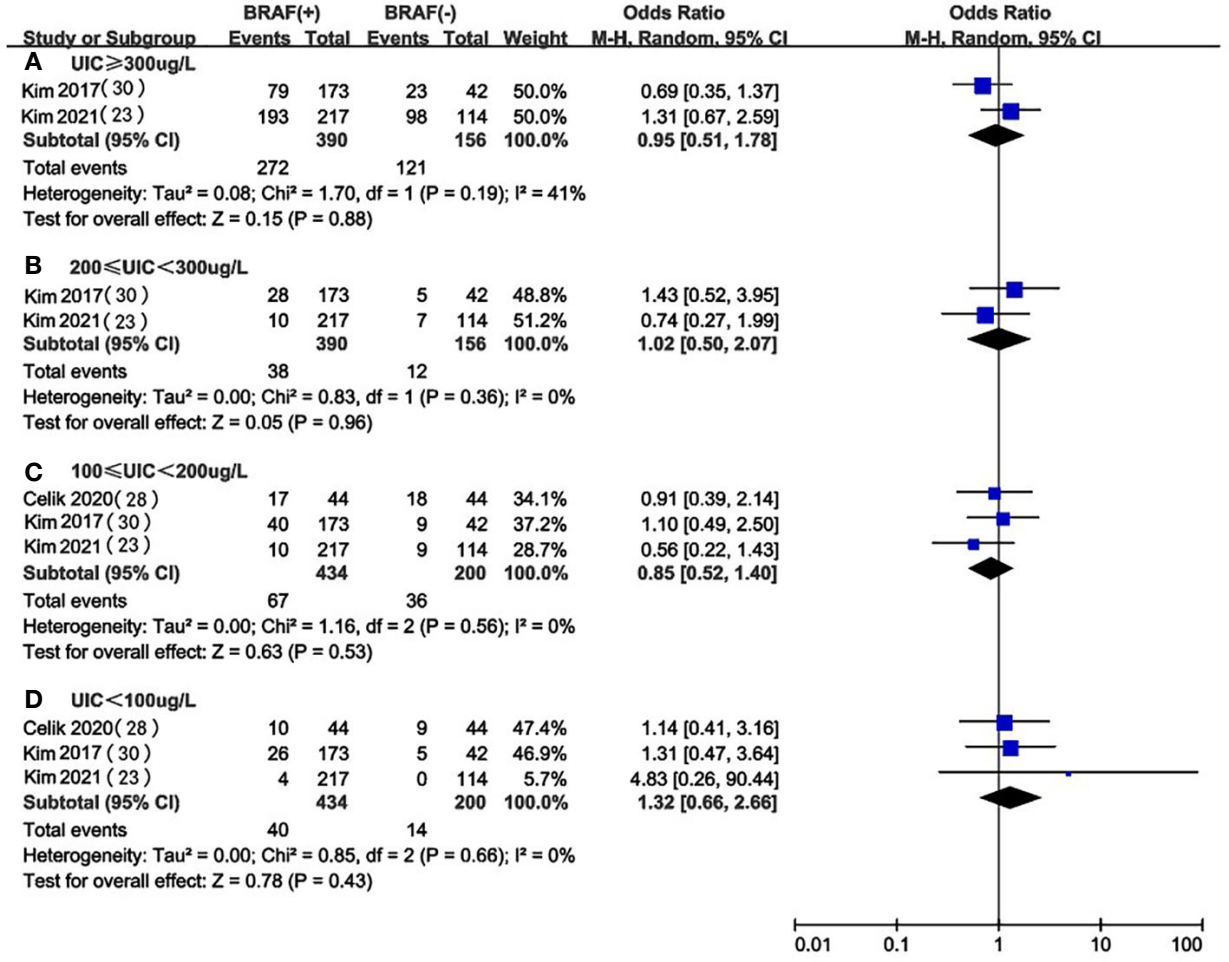
A between-group meta-analysis of UIC in BRAF mutation versus non-BRAF mutation in PTC patients.

We selected 3 articles (23, 25, 29) that compared the association between different levels of UIC and the occurrence of BRAF mutation in PTC patients and did not find significant differences in the levels of UIC between the BRAF mutation positive and negative groups (**Figure 4**). This may suggest that excessive iodine intake (OR 0.95, 95%CI 0.51-1.78, P=0.88, n=546) (**Figure 4A**), above requirements (OR 1.02, 95%CI 0.50-2.07, P=0.96, n=546) (**Figure 4B**), moderate (OR 0.85, 95%CI 0.52-1.40, P=0.53, n=634) (**Figure 4C**) or deficiency (OR 1.32, 95%CI 0.66-2.66, P=0.43, n=634) (**Figure 4D**) do not affect the occurrence of the BRAF mutation in PTC patients.

### UIC in LNM vs. NLNM in PTC patients

**Figure 5:**
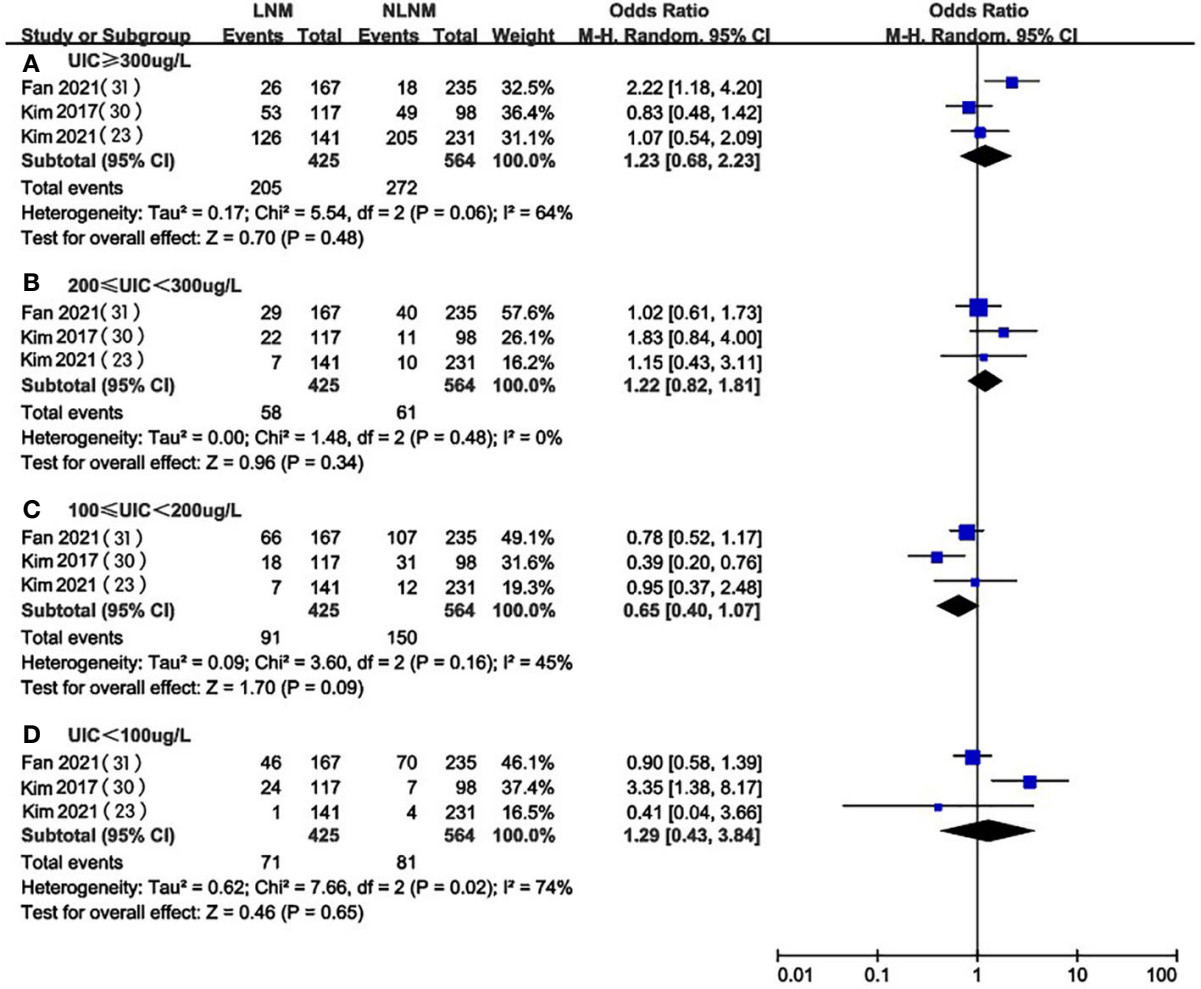
A between-group meta-analysis of UIC in LNM versus NLNM in PTC patients.

Three of the included studies (23, 28, 29) compared UIC levels in PTC patients with and without LNM. We extracted and analyzed the data from these studies and found that there was no significant difference in the prevalence of excessive iodine intake (OR 1.23, 95%CI 0.68-2.23, P=0.48, n=989) (**Figure 5A**), mild overdose (OR 1.22, 95%CI 0.82-1.81, P=0.34, n=989) (**Figure 5B**), moderate (OR 0.65, 95%CI 0.40-1.07, P=0.09, n=989) (**Figure 5C**), or deficiency(OR 1.29, 95%CI 0.43-3.84, P=0.65, n=989) (**Figure 5D**) between PTC patients with or without LNM.

### Subgroup analysis based on the UIC detection methods

**Figure 6:**
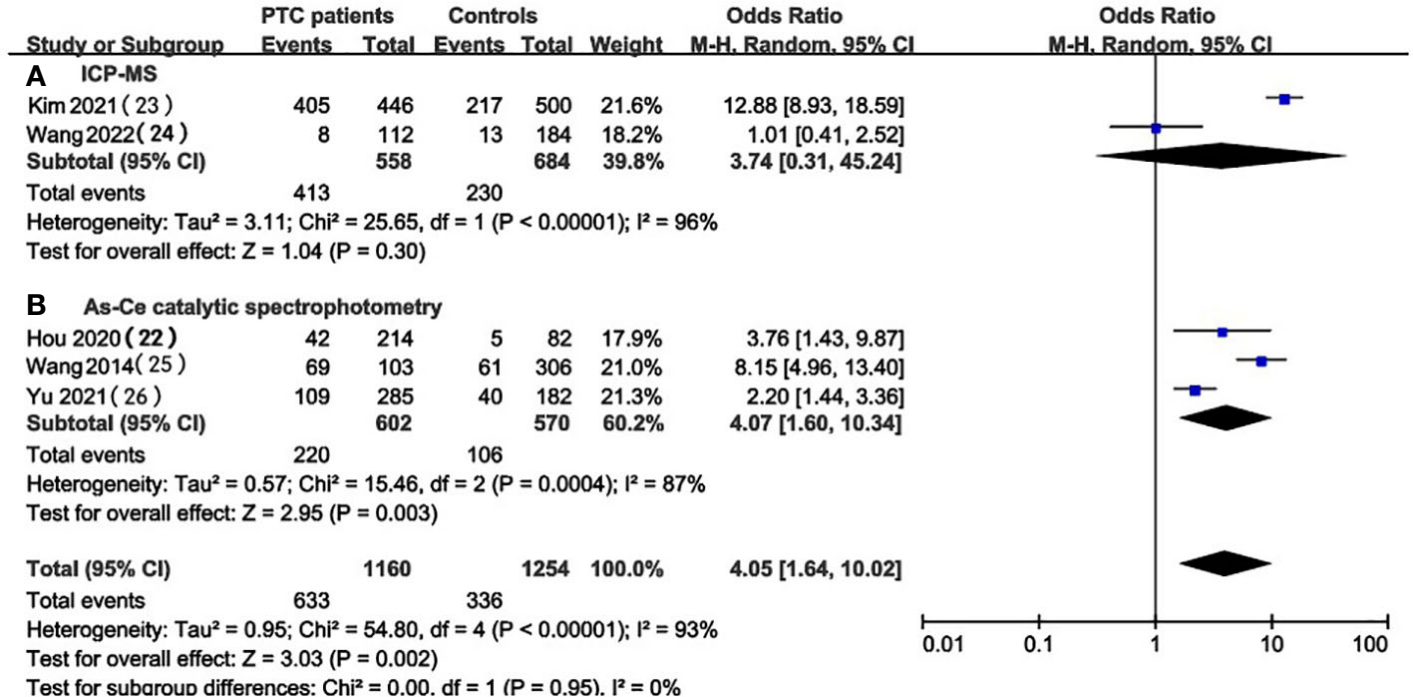
Subgroup meta-analysis of the incidence of high iodine intake (UIC≥300ug/L) in PTC patients versus healthy controls according to UIC detection methods.

**Figure 7:**
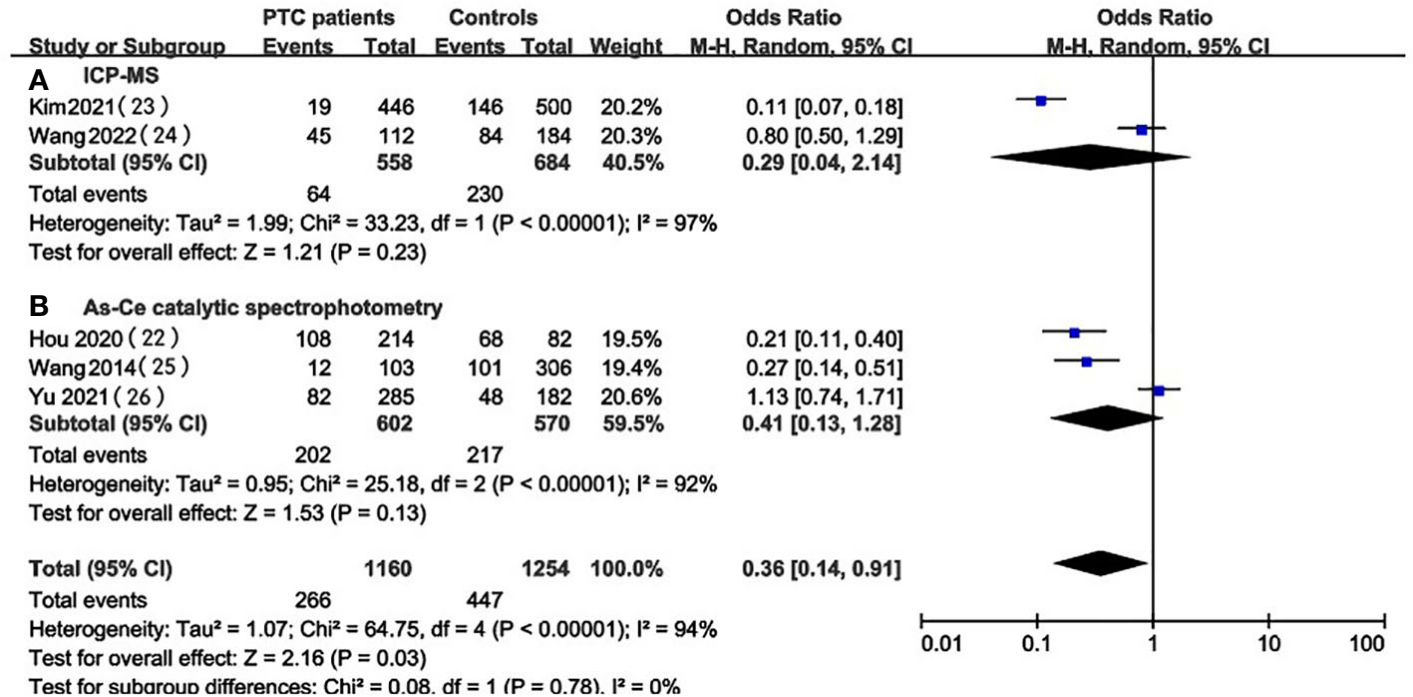
Subgroup meta-analysis of the incidence of adequate iodine intake (100≤UIC<200ug/L) in PTC patients versus healthy controls according to UIC detection methods.

We focused on 5 studies that examined the prevalence of iodine excess in PTC patients and healthy individuals (22, 26, 29–31). A total of two kinds of UIC detection methods were involved in these studies: inductively coupled plasma mass spectrometry (ICP-MS) and As-Ce catalytic spectrophotometry. To investigate whether different UIC detection methods can lead to different results, subgroup analysis was performed based on UIC assays for studies conducted in PTC patients and healthy controls (**Figures 6**, **7**). Despite the stratification by UIC detection methods iodine intake status, the heterogeneity of subgroups was still high. We also verified significant differences between these subgroups. We analyzed the prevalence of excessive iodine intake(UIC≥300ug/L) according to UIC detection methods, which indicated that the prevalence of iodine overdose was significantly higher in PTC patients than in healthy individuals in the As-Ce catalytic spectrophotometry group (OR 4.07, 95% CI 1.60-10.34, P=0.003, n=1172), while the difference in ICP-MS group was not statistically significant (OR 3.74, 95% CI 0.31-45.24, P=0.30, n=1,242) (**Figure 6**). In the group of adequate iodine intake (100≤UIC<200ug/L), we have not found a significant difference between the two groups(As-Ce catalytic spectrophotometry group: OR 0.41, 95% CI 0.13-1.28, P=0.13, n=1172; ICP-MS: OR 0.29, 95% CI 0.04-2.14, P=0.23, n=1,242) (**Figure 7**).

### Sensitivity analysis

To assess the stability of the results, we performed a sensitivity analysis. Each article was successively excluded and a meta-analysis was conducted on the remaining literature, and we observed that the results did not change significantly, which means that the results were stable and reasonable.

## Discussion

Our meta-analysis evaluated 10 case-control studies that used UIC as an alternative indicator for iodine nutrition to investigate its association with the occurrence and invasiveness of PTC. In our results, UIC and the incidence of iodine overdose in PTC patients were significantly higher than those in healthy individuals while there was no significant statistical difference between PTC patients and patients with thyroid adenomas, thyroid nodules, and nodular goiter, which means that the level of UIC in PTC patients was higher than that in healthy controls, but not different from that in patients with other thyroid diseases. Our results support that high urinary iodine or excessive iodine nutrition was related to the occurrence of PTC. In addition, by comparing the UIC of PTC patients with healthy controls, we found that the appropriate dose of UIC has a protective effect on the occurrence of PTC while there was no significant difference in UIC levels between PTC patients and patients with other thyroid diseases. As for iodine deficiency, there was no significant difference observed between the groups.

Iodine, an essential molecule for the synthesis of thyroid hormone and thyroxine, is an important trace element in the human body and mainly accumulates in the thyroid gland and plays an important role in regulating thyroid hormone and metabolic function. Previous studies have indicated that both insufficient and excessive iodine intake can drive thyroid diseases, which implies a U curve relationship (8, 32–34), which means that chronic iodine deficiency or excess can lead to thyroid dysfunction by interfering with homeostasis, proper iodine range will help protect thyroid function while excess or insufficient iodine will promote the occurrence of thyroid diseases. Various studies suggested that iodine intake is probably the influencing factor of PTC (35), our research group also raised the point that excessive iodine intake can have toxic side effects to remind the importance of proper iodine supplementation (36). The viewpoint is consistent with this meta-analysis, which also indicates that moderate iodine intake help protects against PTC, while excess iodine promotes PTC compared with healthy controls. Our results reemphasize the importance of dose-based studies, which may be vital for clarifying the true relationship between iodine status and PTC.

In addition, the combined action of more variables are ought to be considered in the research of iodine and PTC. There are several reasons for the inconsistency between the results of previous studies on iodine and PTC. First of all, different studies used different indicators to reflect iodine nutrition level, such as dietary questionnaires (11, 12, 37–39), regional water iodine (9), salt iodization (10), nail iodine (40), urine iodine (41)and so on. Many of these indicators of iodine nutrient levels are influenced by many factors, such as environment and race, so we suggest the most stable indicators should be considered in future studies. Second, though UIC changes depending on dietary iodine intake, it has been used as a proxy in the population (42). The detection methods of UIC are diverse, the studies included in our meta-analysis involve urinary iodine detection kits, As-Ce catalytic spectrophotometry, Sandell–Kolthoff reaction, ICP-MS, and Sandell–Kolthoff reaction. We have found that the effect of different detection methods varied, so it’s better to use the best measurement to get the most convincing results. In addition, different studies have treated urinary iodine data differently, with many focusing on median UIC differences between case and control groups. Some studies provided creatinine-adjusted and unadjusted UICs for that previous studies have shown that creatinine-adjusted UIC can compensate for the changes in dietary intake and excretion, so it is a better indicator because of its stability (43, 44). We did not include them because there were few eligible studies (29, 41, 45). Considering the choice of the control group, some studies used patients with thyroid nodules or other thyroid diseases as controls. The selection of controls in our analysis distinguished patients with other thyroid disorders, such as thyroid nodules, from healthy participants and found that UIC was significantly different mainly in PTC and healthy controls, while thyroid nodules attenuated this difference. It is suggested that future studies should select appropriate control groups to clarify the true relationship between iodine intake and PTC.

Among various previous inconsistent epidemiological investigations, our analysis provides a clear conclusion that maintaining iodine nutrition in the appropriate range is beneficial for PTC, and it is necessary to avoid excessive iodine intake, which may promote the occurrence of PTC. In the conclusion, our study provides a strong basis for rational iodine supplementation and provides insights for future studies by reviewing existing epidemiological studies.

## Authors contributions

WT is the corresponding author and supervised this work. XQZ is the first and screened the literature with FZ. FZ provided major technical support. QL, RA, and CF assisted in the literature review. DC and XZ checked the data. All authors contributed to the article and approved the submitted version.

## Data availability statement

The original contributions presented in the study are included in the article/supplementary material. Further inquiries can be directed to the corresponding author.

## Funding

This study was supported by The Research Fund for Public Welfare, National Health and Family Planning Commission of China (Grant No. 201402005) and The Clinical Research Fund of Chinese Medical Association (Grant No. 15010010589). The funder had no role in study design, data collection, or analysis, or in the presentation or publication of the results.

## Conflict of interest

The authors declare that the research was conducted in the absence of any commercial or financial relationships that could be construed as a potential conflict of interest.

## Publisher’s note

All claims expressed in this article are solely those of the authors and do not necessarily represent those of their affiliated organizations, or those of the publisher, the editors and the reviewers. Any product that may be evaluated in this article, or claim that may be made by its manufacturer, is not guaranteed or endorsed by the publisher.

## References

[1] Roman BR Morris LG Davies L . The thyroid cancer epidemic, 2017 perspective. Curr Opin Endocrinol Diabetes Obes (2017) 24(5):332–6. doi: 10.1097/MED.0000000000000359 PMC5864110 28692457

[2] Cabanillas ME McFadden DG Durante C . Thyroid cancer. Lancet (2016) 388(10061):2783–95. doi: 10.1016/s0140-6736(16)30172-6 27240885

[3] Peterson E De P Nuttall R . BMI, diet and female reproductive factors as risks for thyroid cancer: a systematic review. PloS One (2012) 7(1):19. doi: 10.1371/journal.pone.0029177 PMC3261873 22276106

[4] Davies L Hoang JK . Thyroid cancer in the USA: current trends and outstanding questions. Lancet Diabetes Endocrinol (2021) 9(1):11–2. doi: 10.1016/S2213-8587(20)30372-7 33220765

[5] Sorrenti S Baldini E Pironi D Lauro A D'Orazi V Tartaglia F . Iodine: Its role in thyroid hormone biosynthesis and beyond. Nutrients (2021) 13(12):4469. doi: 10.3390/nu13124469 34960019 PMC8709459

[6] BÍLek R DvoŘÁKovÁ M GrimmichovÁ T Jiskra J . Iodine, thyroglobulin and thyroid gland. Physiol Res (2020) 69: S225–S36. doi: 10.33549/physiolres.934514 PMC8603726 33094621

[7] Farebrother J Zimmermann MB Andersson M . Excess iodine intake: sources, assessment, and effects on thyroid function. Ann N Y Acad Sci (2019) 1446(1):44–65. doi: 10.1111/nyas.14041 30891786

[8] Sun X Shan Z Teng W . Effects of increased iodine intake on thyroid disorders. Endocrinol Metab (Seoul) (2014) 29(3):240–7. doi: 10.3803/EnM.2014.29.3.240 PMC4192807 25309781

[9] Lv C Yang Y Jiang L Gao L Rong S Darko GM . Association between chronic exposure to different water iodine and thyroid cancer: A retrospective study from 1995 to 2014. Sci Total Environ (2017) 609:735–41. doi: 10.1016/j.scitotenv.2017.07.101 28763670

[10] Harach HR Galindez M Campero M Ceballos GA . Undifferentiated (Anaplastic) thyroid carcinoma and iodine intake in salta, Argentina. Endocr Pathol (2013) 24(3):125–31. doi: 10.1007/s12022-013-9248-9 23666798

[11] Bosetti C Negri E Kolonel L Ron E Franceschi S Preston-Martin S . A pooled analysis of case-control studies of thyroid cancer. VII. cruciferous and other vegetables (International). Cancer Causes Control (2002) 13(8):765–75. doi: 10.1023/a:1020243527152 12420956

[12] Clero E Doyon F Chungue V Rachedi F Boissin J-L Sebbag J . Dietary iodine and thyroid cancer risk in French Polynesia: A case-control study. Thyroid (2012) 22(4):422–9. doi: 10.1089/thy.2011.0173 22280227

[13] Bosetti C Kolonel L Negri E Ron E Franceschi S Maso LD . A pooled analysis of case-control studies of thyroid cancer. VI. fish and shellfish consumption. Cancer Causes Control (2001) 12(4):375–82. doi: 10.1023/a:1011267123398 11456234

[14] Michikawa T Inoue M Shimazu T Sawada N Iwasaki M Sasazuki S . Seaweed consumption and the risk of thyroid cancer in women: the Japan public health center-based prospective study. Eur J Cancer Prev (2012) 21(3):254–60. doi: 10.1097/CEJ.0b013e32834a8042 22414981

[15] Ahn J Lee JH Lee J Baek JY Song E Oh HS . Association between urinary sodium levels and iodine status in Korea. Korean J Intern Med (2020) 35(2):392–9. doi: 10.3904/kjim.2017.375 PMC7061011 29768912

[16] Prete A Paragliola RM Corsello SM . Iodine supplementation: Usage "with a grain of salt". Int J Endocrinol (2015) 2015:312305. doi: 10.1155/2015/312305 25873950 PMC4383497

[17] Zimmermann MB Andersson M . Assessment of iodine nutrition in populations: past, present, and future. Nutr Rev (2012) 70(10):553–70. doi: 10.1111/j.1753-4887.2012.00528.x 23035804

[18] Li Y Teng D Ba J Chen B Du J He L . Efficacy and safety of long-term universal salt iodization on thyroid disorders: Epidemiological evidence from 31 provinces of mainland China. Thyroid (2020) 30(4):568–79. doi: 10.1089/thy.2019.0067 32075540

[19] Eveleigh ER Coneyworth LJ Avery A Welham SJM . Vegans, vegetarians, and omnivores: How does dietary choice influence iodine intake? a systematic review. Nutrients (2020) 12(6):1606. doi: 10.3390/nu12061606 PMC7352501 32486114

[20] Ulisse S Baldini E Lauro A Pironi D Tripodi D Lori E . Papillary thyroid cancer prognosis: An evolving field. Cancers (Basel) (2021) 13(21):5567. doi: 10.3390/cancers13215567 34771729 PMC8582937

[21] Vuong HG Kondo T Oishi N Nakazawa T Mochizuki K Inoue T . Genetic alterations of differentiated thyroid carcinoma in iodine-rich and iodine-deficient countries. Cancer Med (2016) 5(8):1883–9. doi: 10.1002/cam4.781 PMC4898973 27264674

[22] Wang F Wang Y Wang L Wang X Sun C Xing M . Strong association of high urinary iodine with thyroid nodule and papillary thyroid cancer. Tumor Biol (2014) 35(11):11375–9. doi: 10.1007/s13277-014-2397-8 25119588

[23] Kim HJ Park HK Byun DW Suh K Yoo MH Min YK . Iodine intake as a risk factor for BRAF mutations in papillary thyroid cancer patients from an iodine-replete area. Eur J Nutr (2018) 57(2):809–15. doi: 10.1007/s00394-016-1370-2 28258306

[24] Zhao H Li H Huang T . High urinary iodine, thyroid autoantibodies, and thyroid-stimulating hormone for papillary thyroid cancer risk. Biol Trace element Res (2018) 184(2):317–24. doi: 10.1007/s12011-017-1209-6 29164514

[25] Celik M Guldiken S Salt SA Bulbul BY Kucukarda A Can N . Urine iodine excretion in patients with papillary thyroid cancer: Evaluation of the relationship with the presence of a braf mutation. J Elementol (2020) 25(3):1019–28. doi: 10.5601/jelem.2020.25.1.1984

[26] Hou D Xu H Li P Liu J Qian Z . Potential role of iodine excess in papillary thyroid cancer and benign thyroid tumor: A case-control study. Asia Pacific J Clin Nutr (2020) 29(3):603–8. doi: 10.6133/apjcn.202009_29(3).0020 32990621

[27] Huang F Cong W Xiao J Zhou Y Gong M Sun J . Association between excessive chronic iodine exposure and the occurrence of papillary thyroid carcinoma. Oncol Lett (2020) 20(5):189. doi: 10.3892/ol.2020.12051 32952658 PMC7479532

[28] Fan L Tian Q Xiu C Wang F Yuan Z He Q . High iodine nutrition may be a risk factor for cervical lymph node metastasis in papillary thyroid cancer patients. Ann Nutr Metab (2021) 77(2):90–9. doi: 10.1159/000513334 34289482

[29] Kim K Cho SW Park YJ Lee KE Lee D-W Park SK . Association between iodine intake, thyroid function, and papillary thyroid cancer: A case-control study. Endocrinol Metab (2021) 36(4):790–9. doi: 10.3803/EnM.2021.1034 PMC8419609 34376043

[30] Yu Z Yu Y Wan Y Fan J Meng H Li S . Iodine intake level and incidence of thyroid disease in adults in shaanxi province: a cross-sectional study. Ann Transl Med (2021) 9(20):1567. doi: 10.21037/atm-21-4928 34790773 PMC8576709

[31] Wang H Jiang Y Song J Liang H Liu Y Huang J . The risk of perchlorate and iodine on the incidence of thyroid tumors and nodular goiter: a case-control study in southeastern China. Environ Health (2022) 21(1):4. doi: 10.1186/s12940-021-00818-8 34980104 PMC8725411

[32] Kim HJ Kim NK Park HK Byun DW Suh K Yoo MH . Strong association of relatively low and extremely excessive iodine intakes with thyroid cancer in an iodine-replete area. Eur J Nutr (2016) 56(3):965–71. doi: 10.1007/s00394-015-1144-2 26746218

[33] Xiang J Wang X Wang Z Wu Y Li D Shen Q . Effect of different iodine concentrations on well-differentiated thyroid cancer cell behavior and its inner mechanism. Cell Biochem Biophys (2015) 71(1):299–305. doi: 10.1007/s12013-014-0198-8 25120024

[34] Wang B He W Li Q Jia X Yao Q Song R . U-Shaped relationship between iodine status and thyroid autoimmunity risk in adults. Eur J Endocrinol (2019) 181(3):255–66. doi: 10.1530/EJE-19-0212 31252413

[35] Guan H Ji M Bao R Yu H Wang Y Hou P . Association of high iodine intake with the T1799A BRAF mutation in papillary thyroid cancer. J Clin Endocrinol Metab (2009) 94(5):1612–7. doi: 10.1210/jc.2008-2390 19190105

[36] Teng W Shan Z Teng X Guan H Li Y Teng D . Effect of iodine intake on thyroid diseases in China. N Engl J Med (2006) 354(26):2783–93. doi: 10.1056/NEJMoa054022 16807415

[37] Przybylik-Mazurek E Hubalewska-Dydejczyk A Kuzniarz-Rymarz S Kiec-Klimczak M Skalniak A Sowa-Staszczak A . Dietary patterns as risk factors of differentiated thyroid carcinoma. Postepy Higieny I Medycyny Doswiadczalnej (2012) 66:11–5. doi: 10.5604/17322693.974647 22371400

[38] Sangsefidi ZS Ghafouri-Taleghani F Zakavi SR Norouzy A Kashanifar R Pourbaferani R . Major dietary patterns and differentiated thyroid cancer. Clin Nutr ESPEN (2019) 33:195–201. doi: 10.1016/j.clnesp.2019.05.015 31451261

[39] Truong T Baron-Dubourdieu D Rougier Y Guenel P . Role of dietary iodine and cruciferous vegetables in thyroid cancer: a countrywide case-control study in new Caledonia. Cancer Causes Control (2010) 21(8):1183–92. doi: 10.1007/s10552-010-9545-2 PMC3496161 20361352

[40] Horn-Ross PL Morris JS Lee M West DW Whittemore AS McDougall IR . Iodine and thyroid cancer risk among women in a multiethnic population: The bay area thyroid cancer study. Cancer Epidemiol Biomarkers Prev (2001) 10(9):979–85. 11535551

[41] Zeng Z Li K Wang X Ouyang S Zhang Z Liu Z . Low urinary iodine is a protective factor of central lymph node metastasis in papillary thyroid cancer: a cross-sectional study. World J Surg Oncol (2021) 19(1):208. doi: 10.1186/s12957-021-02302-6 34253203 PMC8276512

[42] Vejbjerg P Knudsen N Perrild H Laurberg P Andersen S Rasmussen LB . Estimation of iodine intake from various urinary iodine measurements in population studies. Thyroid (2009) 19(11):1281–6. doi: 10.1089/thy.2009.0094 19888863

[43] Kim HK Lee SY Lee JI Jang HW Kim SK Chung HS . Usefulness of iodine/creatinine ratio from spot-urine samples to evaluate the effectiveness of low-iodine diet preparation for radioiodine therapy. Clin Endocrinol (2010) 73(1):114–8. doi: 10.1111/j.1365-2265.2009.03774.x 20050860

[44] Caldwell KL Maxwell CB Makhmudov A Pino S Braverman LE Jones RL . Use of inductively coupled plasma mass spectrometry to measure urinary iodine in NHANES 2000: comparison with previous method. Clin Chem (2003) 49(6 Pt 1):1019–21. doi: 10.1373/49.6.1019 12766019

[45] Lee J . Case-control study of papillary thyroid carcinoma on urinary and dietary iodine status in south Korea. Ann Surg Oncol (2018) 25(1):S17–S8. doi: 10.1245/s10434-018-6349-1 29067516

